# Use of laboratory and administrative data to understand the potential impact of human parainfluenza virus 4 on cases of bronchiolitis, croup, and pneumonia in Alberta, Canada

**DOI:** 10.1186/s12879-016-1748-z

**Published:** 2016-08-11

**Authors:** Sumana Fathima, Kimberley Simmonds, Jesse Invik, Allison N. Scott, Steven Drews

**Affiliations:** 1 Provincial Laboratory for Public Health (ProvLab), Calgary, AB Canada; 2 Alberta Health, Edmonton, AB Canada; 3 University of Calgary, Calgary, AB T2N 1N4 Canada; 4 Pathology and Laboratory Medicine, University of Alberta, Edmonton, AB Canada; 5 Provincial Laboratory for Public Health (ProvLab), Edmonton, AB 2B1.03 WMC Canada; 6 University of Alberta Hospital, 8440-112 St, Edmonton, AB T6G 2J2 Canada

**Keywords:** Parainfluenza, Bronchiolitis, Croup, Pneumonia, Descriptive, Respiratory, Virus

## Abstract

**Background:**

Human Parainfluenza Virus (hPIV) causes severe respiratory illness in infants and adults. Our study describes the association of hPIV1–4 with bronchiolitis, croup, and pneumonia using retrospective laboratory, administrative and public health data. Due to issues including the historic lack of hPIV4 in some commercial respiratory virus panels, the description of the impact of hPIV4 on croup, bronchiolitis, and pneumonia at population levels has often been limited. This study will use routine clinical laboratory data, and administrative data to provide a preliminary description of the impact of hPIV4 on these diseases in our population.

**Methods:**

A three year cohort of patients positive for hPIV was linked with data from physician visits and hospital admissions to define cases and hospitalization status. International Classification of Disease (ICD-9) codes were used to determine if cases had croup, bronchiolitis, and pneumonia. We also looked at differences in hospitalization status, age and gender among hPIV1–4. All statistical analysis was done using SPSS (Version 19.0.0, IBM Corp© 2010) and Graphpad Prism V6 (GraphPad Software, Inc., 2012).

**Results:**

Only hPIV1 and hPIV4 specimens had positivity rates greater than 5 % of all specimens sent for respiratory virus panel testing. hPIV1 exhibited a biennial pattern while the pattern for hPIV3 was less interpretable due to lower positivity rates. Circulation patterns for hPIV2 and hPIV4 were not assessed due to the low positivity rates of theses specimens. From 2010 to 2013, there were 2300 hPIV cases with hPIV3 (46 %) being the most common, followed by hPIV1 (27 %), hPIV4 (16 %) and hPIV2 (11 %). The median age was 2 years for all hPIV types. Males were slightly greater than females for hPIV1 and hPIV2, with an equal distribution for hPIV3 and slightly more females than males for hPIV4. hPIV1 and hPIV2 had the highest or proportion of croup while hPIV3 and hPIV4 had the highest proportion of pneumonia. Within hPIV4 cases, distributions of diseases were; pneumonia (21 %, 95 % CI 17.1–25.7), bronchiolitis (18 %, 95 % CI 14.3–22.5), croup (2 %, 95 % CI 0.8–3.9), mixed illness of any of pneumonia, bronchiolitis or croup (4 %, 95 % CI 2.5–7.0) or other respiratory diseases (54 %, 95 % CI 49.1–59.6).

**Conclusions:**

We used laboratory and administrative data to undertake a descriptive analysis of the association of hPIV1–4 with croup, bronchiolitis and pneumonia. hPIV4 appears to be more associated more with bronchiolitis and pneumonia and less with croup in our population.

## Background

Respiratory illness due to viral infections represents a significant burden on the healthcare system and our jurisdiction is highly impacted by respiratory viruses from later summer until early spring [1]. Human Parainfluenzavirus (hPIV) is a single stranded RNA virus belonging to *Paramyxoviridae* family, and all four types are a significant cause of respiratory illness, in infants and elderly [2]. hPIVs cause upper respiratory tract illness (URTI) such as colds, otitis media, and pharyngitis or lower respiratory tract illness (LRTI) such as croup, bronchiolitis and pneumonia [3–5]. In adults, the infection tends to stay in the upper respiratory tract; however morbidity in children under the age of five can be severe, with hospitalizations due to pneumonia, croup, and bronchiolitis [2]. The four types of parainfluenza differ in their clinical outcomes. hPIV1 and 2 have been associated with croup in children (acute laryngotracheobronchitis) [6], while hPIV3 has been described in bronchiolitis and pneumonia [7].

Although hPIV4 has been described in studies using laboratory developed tests, it has not often been included in some commercial respiratory viral panels and so large scale systematic surveillance may not have included hPIV4 due to regulatory issues [8]. We note that there is already some work suggesting an association of hPIV4 infections with pneumonia [9] and some speculation that hPIV4 infections might resemble hPIV3 infections [10, 11]. However, studies on the impact of HPIV4 on specific diseases have been limited to locations that able to overcome this limitation and integrate testing into diagnostic algorithms and that studies may not have focused on whole populations. In contrast, our laboratory provides centralized testing for our Province (Population 4.1 Million) and was one of the early adopters of a diagnostic that could differentiate hPIV4 from hPHIV1-3 on a population level over a period of several years [10, 12, 13]. Due to a number of historical factors, we also undertake extremely high levels of respiratory virus testing a year with this technology (approximately 30,000 specimens a year), for a variety of patient populations which allows us extensive coverage of a large population from a variety of settings. Similarly, our publically funded health care system allows us to link billing codes indicating suspect diagnosis to patient laboratory information.

We also wanted to determine whether hPIV4 infections exhibited similar patterns of prevalence to other hPIV types. Variability of temporal and seasonal patterns of prevalence (annual, biannual, biennial) for hPIV types have been described in the literature but the data for hPIV4 has been relatively less described [14]. hPIV4 infections in other populations have been described as occurring in a year round distribution, with biennial peaks in odd-numbered years and a spectrum of disease [10]. In contrast prevalence patterns for the other hPIV types 1–3 may vary. Some studies in the United States have also shown that hPIV1 occurs in the fall of odd numbered years and hPIV2 occurs in even-numbered years [15]. While hPIV3 is observed year round, it tends to peak primarily in the spring and early summer [16] or might have a biannual pattern of prevalence [10]. HPIV2 has shown to have varied seasonal distribution, often occurring in multiple seasons and characterization of seasonality for HPIV2 is further confounded by the low prevalence of this virus [17, 18].

Therefore, the purpose of the study is to use routine clinical laboratory data, and administrative data collected by public health to provide a preliminary description of the impact of hPIV4 on croup, bronchiolitis and pneumonia in our population. We intended to do this by comparing the distribution of ICD9 codes associated with these diseases between different hPIV types. We also wanted to provide a better understanding of the circulation patterns of PIV4 over time in our province.

## Methods

Alberta is the fourth largest province in Canada with a population of 4.1 million [19]. Alberta has universal health care; as such, all inpatient and outpatient visits to physicians, emergency departments, and hospitals for all Alberta residents are billed to the Alberta Ministry of Health. All Alberta residents are issued a unique Public Health Number (PHN) at birth or upon moving to the province. Physicians are required to include this identifier and the diagnosis of the patient when billing for inpatient or outpatient services.

The Provincial Laboratory for Public Health (ProvLab) is a major diagnostic lab in the province of Alberta. All inpatient and outpatient respiratory samples throughout the province are labeled with the patient’s PHN and sent to ProvLab for laboratory testing and the resulting test results are exported and stored in a database. For the time period in this analysis, the determination to order respiratory virus testing was left to the discretion of the ordering physician and there were not recommendations provided on when a respiratory virus panel should be ordered. In combining the ProvLab and Ministry of Health data sources, it is possible to determine the organism an Alberta resident was infected with, whether they were seen as an inpatient or an outpatient, and whether they were hospitalized. The study utilized this capability to describe the epidemiology of hPIV1–4 in the population of Alberta.

During the study period, all respiratory specimens were routinely tested for influenza A and influenza B using a real-time reverse transcriptase-real time polymerase chain reaction (RT-PCR) assay as previously described [1]; if found negative, they were tested by the XTAG® Respiratory Viral Panel (RVP, Luminex, Austin, TX, USA) for several other common respiratory pathogens including, hPIV1–4 [20]. Clinical respiratory samples from Alberta residents received at the ProvLab between November 1, 2010 and December 31, 2013 that tested positive for hPIV1–4 were eligible for the study. Specimen positivity rates were calculated by determining the number of positive hPIV1–4 specimens detected versus the number of specimens that were tested by the RVP panel.

As patients could have multiple samples sent for testing, only the first positive sample for an individual within a 365 day period for a specific type of hPIV was allowed in the study. Subsequent positive tests were excluded within the 365 day period, unless they were positive for a different type of hPIV, at which time they were considered a different case of disease. This period of time was chosen as we wanted to ensure that the values we presented were conservative estimates of the number of cases diagnosed during the study period. Cases that were co-infected with more than one type of hPIV or co-infected with another virus (such as rhino-enterovirus or respiratory syncytial virus [RSV].) were also excluded from the analysis to prevent the confounding effect of other viruses that may also be associated with croup, bronchiolitis and pneumonia [21].

Positive cases of hPIV were deterministically linked to administrative data using the PHN. The physician claims data in the Supplemental Enhanced Service Event (SESE) database was used to provide the World Health Organization (WHO) International Classification of Diseases 9 Codes (ICD 9) [22] associated with the physician visit and associated hIPV laboratory result. Three ICD 9 codes were extracted from the SESE database along with patient demographic characteristics. There were often multiple ICD9 codes, for the non-croup, non-bronchiolitis, and non-pneumonia case group. For this group frequencies of other disease codes included; “infectious diseases” (001–139), “ill defined condition” (780–799), acute respiratory infections (460–466); other diseases of the respiratory system (467–519), diseases of the ear and mastoid process (380–389), other diseases of the central nervous system and sense organs (320–379).

Physician claims data is housed in the Supplemental Enhanced Service Event System (SESE database); inpatient hospitalizations, emergency department visits, and outpatient clinic data is housed in the Morbidity and Ambulatory Care Abstracting Reporting (MACAR) system, which feeds into the Canadian Institute for Health Information’s (CIHI) Discharge Abstract Database (DAD) and National Ambulatory Care Reporting System (NACRS). [23]. The hospitalization data provided information on whether the case was hospitalized and the duration of hospitalization.

These databases were utilized to determine demographic information, and to determine if a hPIV case was diagnosed with croup, pneumonia, or bronchiolitis within 14 days of their positive parainfluenza specimen. Table 1 lists the ICD-9 codes used to define each diagnosis. “Single diagnosis” was defined as hPIV cases that had one event of croup, bronchiolitis or pneumonia. “Mixed diagnoses” were defined as hPIV cases diagnosed with two or more events (i.e. croup and pneumonia).

**Table 1:** Description of ICD-9 codes used to diagnose hPIV laboratory tested cases with public health administrative databases

Diagnosis	ICD-9 diagnostic codes
Croup	464, 464.1, 464.4, 464.3, 464.4
Bronchiolitis	466, 466.0, 466.1
Pneumonia	480–486 (inclusive)

Descriptive statistics regarding demographic characteristics and clinical diagnosis of hPIV1–4 cases were performed. We also described the distribution of hPIV cases over time. Proportions, means, medians were performed using SPSS (Version 19.0.0, IBM Corp© 2010). Graphpad Prism (V6, (GraphPad Software, Inc., 2012) was used to obtain 95 % confidence interval (CI) of proportions. Fisher’s exact and Chi- square tests were utilized to test for statistical significance.

## Results

Identification of positive hPIV cases was carried out as follows. A total of 55,112 specimens were tested for hPIV and included in the study. 63.5 % (51,888/81,587) specimens tested negative for hPIV, leaving 3224 positive specimens. Negatives were not included in Table 2 because the point of this analysis was to compare the distribution of ICD9 codes for croup, bronchiolitis, and pneumonia with specific hPIV types. Of those 3224 specimens, 316 were considered duplicate (i.e. based on case definition), leaving 2908 unique cases of hPIV. Twenty five cases were unable to be linked to the SESE and MACAR databases due to invalid PHN’s or other personal identifiers and were therefore excluded from study at this point. We were left with 2883 cases of hPIV that were linked to SESE and MACAR. Furthermore we excluded 19 cases that had mixed hPIVs, and 564 cases that were co-infected with other respiratory viruses leaving a total of 2300 unique hPIV cases over a three year period.

**Table 2:** Descriptive analysis of all combined hPIV cases laboratory and public health linked cases 2010–2013

	All cases (n, %) *N* = 2300
hPIV1	617 (27)
hPIV2	252 (11)
hPIV3	1067 (46)
hPIV4	364 (16)
Median age years (range)	2 (0.01–103)
Age	
0–1 years	1049 (46)
2–4 years	386 (17)
5–8 years	154 (7)
9–11 years	32 (1.4)
12–17 years	52 (2.3)
18–64 years	367 (16)
65 + years	259 (11)
Unknown	1 (0.04)
Gender	
Male	1196 (52)
Female	1090 (47)
Unknown	2 (1)
Non hospitalized (Family Doc or ER visit with no inpatient stay)	1332 (58)
Hospitalized	968 (42)
Croup	248 (11)
Bronchiolitis	305 (13)
Pneumonia	377 (16)
Mixed cases that have Croup Bronchiolitis and Pneumonia	103 (5)
Other clinical or respiratory disease	1267^a^ (55)

51,888 negative specimens were excluded from analysis as the goal of this study was to compare the distribution of ICD9 codes for croup, bronchiolitis and pneumonia for hPIV1–4

^a^ These cases had a respiratory disease ICD-9 code that was not specific for croup, bronchiolitis or pneumonia. Out of 1267 cases, 835 (63 %) were non-hospitalized

As in Table 2, hPIV4 was the third most common hPIV type making up 16 % of all hPIV cases. Out of all the cases, hPIV3 was the most common (46 %) type followed by hPIV1 (27 %). hPIV2 was the least common of all the hPIV cases (11 %). The median age was 2 years (range: 0.01–103 years) for all hPIV types. Males were slightly greater than females for hPIV1 and hPIV2, with an equal distribution for hPIV3 and slightly more females than males for hPIV4. Greater than 50 % of cases were non-hospitalized (Table 2). Over 50 % of cases were associated with ICD-9 codes related to other respiratory diseases that were not croup, bronchiolitis and pneumonia. As shown in Table 3, 95 % confidence intervals of the hPIV types for hospitalized cases all overlapped which indicated to use that were no able to comfortably infer a dominance of any one type in this population. The same inability to infer a dominant type of hPIV was also found with the non-hospitalized group. hPIV1 and hPIV2 had the highest percentages of croup while hPIV3 and hPIV4 had the highest percentages of pneumonia. Within hPIV4 cases, distributions of diseases were; pneumonia (21 %, 95 % CI 17.1–25.7), bronchiolitis (18 %, 95 % CI 14.3–22.5), croup (2 %, 95 % CI 0.8–3.9), mixed illness of any of pneumonia, bronchiolitis or croup (4 %, 95 % CI 2.5–7.0) or other respiratory diseases (54 %, 95 % CI 49.1–59.6).

**Table 3:** Descriptive analysis of hPIV laboratory and public health linked cases depending on type

	hPIV1 (*N* = 617) n (%)	95 % CI	hPIV2 (*N* = 252) n (%)	95 % CI	hPIV3 (*N* = 1067) n (%)	95 % CI	hPIV4 (*N* = 364) n (%)	95 % CI
Hospitalized^a^	259 (42)	38.0–45.9	107 (42)	36.2–48.8	436 (41)	37.9–43.9	166 (46)	40.4–50.8
Non-Hospitalized	358 (58)	54.0–61.9	145 (58)	51.1–63.7	631 (59)	56.1–62.0	198 (54)	49.1–59.5
<2 years^b^	285 (46)	42.2–50.2	101 (40)	34.0–46.4	505 (47)	44.3–50.4	158 (44)	38.1–48.5
2–4 years	141 (23)	19.6–26.4	40 (16)	11.6–21.0	146 (14)	11.7–15.9	59 (16)	12.6–20.5
5–8 years	62 (10)	7.8–12.7	36 (14)	10.2–19.2	28 (3)	1.8–3.8	28 (8)	5.2–11.0
9–11 years	7 (1)	0.5–2.3	12 (5)	2.5–8.2	9 (0.8)	0.4–1.4	4 (1.1)	0.3–2.8
12–17 years	10 (2)	0.8–3.0	6 (2)	0.9–5.1	24 (2.2)	1.4–3.3	12 (3.3)	1.7–5.7
18–64 years	78 (13)	10.1–15.5	33 (13)	9.2–17.9	183 (17)	14.9–19.5	73 (20)	16.1–24.6
65 + years	34 (6)	3.8–7.6	23 (9)	5.9–13.4	172 (16)	14.0–18.5	30 (8.2)	5.6–11.6
Unk	0 (0)	0.0–0.6	1 (0.4)	0.0–2.2	0 (0)	0.0–0.3	0 (0)	0.0–1.0
Male^c^	343 (56)	51.6–59.6	138 (55)	48.4–61.0	538 (50)	47.4–53.5	177 (49)	43.4–53.9
Female	270 (44)	39.8–47.8	113 (45)	38.6–51.2	523 (49)	46.0–52.1	184 (51)	45.3–55.8
Unknown	4 (0.65)	0.2–1.7	1 (0.39)	0.0–2.2	6 (0.6)	0.2–1.2	3 (0.8)	0.2–2.4
Croup^d^	133 (22)	18.4–25.0	47 (19)	14.0–24.0	61 (6)	4.4–7.3	7 (2)	0.8–3.9
Pneumonia^e^	74 (12)	9.5–14.8	33 (13)	9.2–17.9	193 (18)	15.8–20.5	77 (21)	17.1–25.7
Bronchiolitis^f^	64 (11)	8.1–13.1	27 (11)	7.2–15.2	148 (14)	11.9–16.1	66 (18)	14.3–22.5
Mixed^g^	33 (5)	7.4–37.0	13 (5)	8.7–28.0	41 (4)	2.8–5.2	16 (4)	2.5–7.0
Other respiratory disease	313 (50)	46.7–54.7	132 (52)	46.0–58.7	624 (58)	55.5–61.5	198 (54)	49.1–59.6

Mixed means case has any combination of croup, pneumonia and bronchiolitis

^a^ NS hospitalized vs non-hospitalized for PIV type: *P* = 0.4717; ^b^ NS age for PIV type, *P* = 0.307; ^c^ NS male vs female for PIV type, *P* = 0.0847

^d^ Chi-square 148.82, df = 3, *P* < 0.001; ^e^ Chi-square 24.13, df = 3, *P* < 0.001; ^f^ Chi-square 13.75, df = 3, *P* = 0.0033; ^g^ Chi-square 10.52, df = 3, *P* = 0.0146

The association of PIV1-4 was also assessed for other disease codes for the non-croup, non-bronchiolitis, and non-pneumonia cases (*n* = 1267). As there were multiple ICD9 codes, for each non-case in this group, frequencies of other disease codes were described. Many of these were relatively non-specific. Frequencies for “infectious diseases” (001–139) for each PIV type were; PIV1 [52/313, 16.61 %), PIV2 (30/132, 22.72 %), PIV3 (78/624, 12.50 %), and PIV4 (23/198. 11.62 %). Frequencies for “ill defined condition” (780–799) for PIV types were; PIV1 (137/313, 43.77 %), PIV2 (50/132, 37.88 %), PIV3 (252/624, 40.38 %), and PIV4 (87/198, 43.94 %). Frequencies for acute respiratory infections (460–466) for PIV types were; PIV1 (114/313, 36.42 %), PIV2 (38/132, 28.79 %), PIV3 (162/624, 25.96 %), and PIV4 (54/198, 27.27 %). Frequencies for other diseases of the respiratory system (467–519) for PIV types were; PIV1 (58/313, 18.53 %), PIV2 (26/132, 19.70 %), PIV3 (159/625, 25.44 %), an PIV4 (40/198, 20.20 %). Frequencies of, diseases of the ear and mastoid process (380–389) for each PIV type were; PIV1 (13/313, 4.15 %), PIV2 (4/132, 3.03 %), PIV3 (27/624, 4.32 %), and PIV4 (7/198, 3.53 %). Frequencies for other diseases of the central nervous system and sense organs (320–379) for each PIV type were; PIV1 (17/313, 5.43 %), PIV2 (5/132, 3.79 %), PIV3 (56/624, 8.97 %), and PIV4 (14/198, 7.07 %). No significant difference was seen between PIV type and diseases of the “ear and mastoid” (Chi-square, *p* = 0.89) nor for other diseases of the “nervous system and sense organs (Chi-square, *p* = 0.0724).

**Fig. 1:**
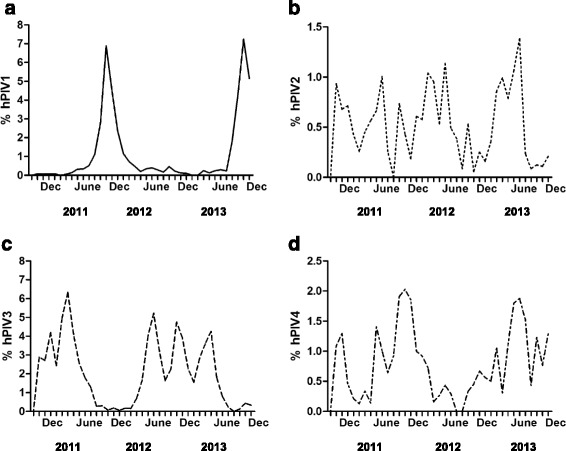
Temporal distribution of positive (**a**) hPIV1 specimens, (**b**) hPIV2 specimens, (**c**) hPIV3 specimens and (**d**) hPIV4 specimens. Only hPIV1 and hPIV3 specimens had peak rates of positivity greater than 5 % when compared to total numbers of specimens tested by RVP. Rates of hPIV2 and hPIV4 specimen positivity were consistently below 1.5 and 2.5 % respectively and were not analyzed for seasonal or yearly trends

As in Fig. 1, only hPIV1 and hPIV3 specimens had peak positivity rates greater than 5 %. The peak patterns for hPIV1 were more defined and there appeared to be two winter peaks each separated by a one year period with no hPIV1 peak in what can be described as a biennial pattern (Fig. 1a). The peak patterns for hPIV3 (Fig. 1c) were less defined with one major peak in the winter period of 2010–2011, which occurred out of cycle with the hPIV1 peaks. Latter peaks for hPIV3 were less easy to interpret due to lower positivity rates that hovered around the five percent positivity level. Temporal peak patterns for hPIV2 (Fig. 1b) and hPIV4 (Fig. 1d) were not assessed as these did not peak above 5 % positivity.

## Discussion

This study uses administrative and hospitalization codes to link clinical laboratory data from hPIV1–4 cases to a clinical diagnosis (e.g. croup, bronchiolitis, pneumonia) and to determine patient setting (hospital or non-hospitalized). ICD-9 codes have been shown to be valid markers of a true history of pneumonia and can be used to successfully identify infection-related conditions in epidemiologic studies [24]. Previous epidemiologic studies in Canada have used ICD-9 codes to understand patterns of croup. These codes have also been used to analyze bronchiolitis in epidemiologic studies in a variety of settings and over time [25–28]. ICD-9 codes have also been utilized to characterize croup cases on a population basis in other studies [29, 30]. The use of these data sets allows for analysis of general trends of illness at population levels in a manner that could not be accomplished with later more detailed chart reviews and surveys of electronic health records. We have also previously used hospitalization diagnosis codes in our jurisdiction to understand patient location trends on a population level [20]. However, we hope that linking multiple databases will lead to additional studies that can provide a greater understanding of the role that that these hPIV types play in acute respiratory infections. Using administrative and clinical data we were able to study a largely pediatric patient population with nearly three quarters of all hPIV-positive patients age 12 or younger, and 42 % of hPIV cases as being hospitalized.

This work sheds light on some basic characteristics of hPIV4 infection at a larger population level. Of all the hPIV types, the clinical and epidemiologic presentation of hPIV4 on a large population level have been the least studied [10], although hPIV4 has been described as a cause of acute influenza-like-illness (ILI) in both adults and children in a variety of settings [13, 31–34]. This increasing body of work contrasts to earlier studies which identified hPIV4 as being associated with mild disease [35] or predominantly upper respiratory tract infections [36]. Instead, our descriptive analysis aligns more with recent studies describing hPIV4 in acute lower respiratory tract infections in pediatric patients [36, 37], as an etiologic agent in hospitalized cases of community acquired pneumonia [38], or being associated with bronchiolitis. This difference in disease patterns may be due to the broader sampling from our study (e.g. the whole jurisdiction) including acute care and tertiary care facilities instead of community settings. Thus, we may be identifying a physician bias towards testing patients who are more ill and we maybe cases who have a greater severity of illness and are more likely to be evaluated.

One recent study by Frost et al., recently did an excellent job at describing the epidemiology of hPIV4 in hospitalized pediatric patients in Colorado, USA. That study did not identify any cases of hPIV4 associated with croup [10]. In contrast, our study found that seven percent of hPIV4 cases were diagnosed with croup. This may be accounted for by the fact that our study was a large population-based study over an extended time period and large geographic area and included both pediatric and adult, and hospitalized and non-hospitalized cases.

Although some differences in type-associate disease profiles are seen in the study against previous work, the seasonality of hPIV4 was not determined in this study due to low peak positivity rates of hPIV4 specimens. Peak hPIV4 activity has been described in the late autumn and early winter [39]. Depending on the study both hPIV4 and hPIV2, have either annual or biennial circulation patterns [10]. In contrast a biennial circulation trend was observed for the hPIV1 cases and peak hPIV3 activity was offset from peak hPIV1 activity by one year [6, 15, 39–43]. Published and unpublished differences in temporal circulation patterns between studies is not unexpected, as multiple host and environmental factors impact on the seasonal distributions of these viruses [44].

There are several limitations to this study. First, the analysis does not describe the differences between the two subtypes of hPIV4 [45], and the work excludes co-infections of hPIV types or co-infections with other respiratory viruses. The study also does not attempt to understand the deeper clinical histories of patients, including factors such as immunocompromised status or transplant that might be associated with acute respiratory infections by specific hPIV types. The lack of a standardized case definition, reinfection rates, or maximum shedding periods for parainfluenza impact comparisons between studies [43, 46–48]. While 1 year may be high, we felt this was justified as we wanted to ensure that the values we presented were conservative estimates of the number of cases diagnosed during the study period, and that the estimates of hospitalization were not overestimated by the potential inclusion of very ill patients being tested multiple times for the same illness. However, we are reassured that only 316 of 3224 specimens were excluded from the study as duplicates. Finally, this is an analysis of large sets of administrative and laboratory data which may yield different conclusions than clinical studies using chart reviews. Although there is the possibility of errors in diagnostic codes, the authors feel that the diagnostic codes for emergency department and inpatient data are valid and reliable due to the rigorous training and review that the Canadian Institute for Health Information (CIHI) imposes [49, 50].

## Conclusions

This study suggests that the distribution clinical illnesses may be different between hPIV4 and hPIV1. While the proportion of hPIV4 cases diagnosed with croup was statistically significantly lower than hPIV1 cases, hPIV1 cases were statistically significantly more likely to be diagnosed with pneumonia and bronchiolitis. While the results did not attain statistical significance, a similar trend was seen with hPIV2. The difference in the distribution of clinical diagnoses in hPIV types suggests that in the future it may be worthwhile to support hPIV type specific patient management strategies.

## Abbreviations

CIHI, Canadian Institute for Health Information; DAD, Discharge Abstract Database; hPIV, human parainfluenza virus; ICD-9, international classification of diseases-9; ILI, influenza-like illness; LRTI, lower respiratory tract infection; MACAR, morbidity and ambulatory care reporting database; NACRS, National Ambulatory Care Reporting System; PHN, public health number; RSV, respiratory syncytial virus; RT-PCR, real-time reverse transcriptase-real time polymerase chain reaction; SESE, Supplemental Enhanced Service Event System; URTI, upper respiratory tract infection; WHO, World Health Organization
